# Experimental infection of Foxes with European bat *Lyssaviruses *type-1 and 2

**DOI:** 10.1186/1746-6148-5-19

**Published:** 2009-05-19

**Authors:** Florence Cliquet, Evelyne Picard-Meyer, Jacques Barrat, Sharon M Brookes, Derek M Healy, Marine Wasniewski, Estelle Litaize, Mélanie Biarnais, Linda Johnson, Anthony R Fooks

**Affiliations:** 1 WHO Collaborating Centre for Research and Management in Zoonoses Control, OIE Reference Laboratory for Rabies, Community Reference Laboratory for Rabies, Community Reference Laboratory for Rabies Serology, AFSSA Malzeville, France; 2 WHO Collaborating Centre for the Characterization of Rabies and Related Rabies Viruses, Rabies and Wildlife Zoonoses Group, Veterinary Laboratories Agency, Weybridge, UK

## Abstract

**Background:**

Since 1954, there have been in excess of 800 cases of rabies as a result of European Bat *Lyssaviruses *types 1 and 2 (EBLV-1, EBLV-2) infection, mainly in Serotine and Myotis bats respectively. These viruses have rarely been reported to infect humans and terrestrial mammals, as the only exceptions are sheep in Denmark, a stone marten in Germany and a cat in France. The purpose of this study was to investigate the susceptibility of foxes to EBLVs using silver foxes (*Vulpes vulpes*) as a model.

**Results:**

Our experimental studies have shown that the susceptibility of foxes to EBLVs is low by the intramuscular (IM) route, however, animals were sensitive to intracranial (IC) inoculation. Mortality was 100% for both EBLV-1 (~4.5 logs) and EBLV-2 (~3.0 logs) delivered by the IC route. Virus dissemination and inflammatory infiltrate in the brain were demonstrated but virus specific neutralising antibody (VNA) was limited (log(ED_50_) = 0.24–2.23 and 0.95–2.39 respectively for specific EBLV-1 and EBLV-2). Foxes were also susceptible, at a low level, to peripheral (IM) infection (~3.0 logs) with EBLV-1 but not EBLV-2. Three out of 21 (14.3%) foxes developed clinical signs between 14 and 24 days post-EBLV-1 infection. None of the animals given EBLV-2 developed clinical disease.

**Conclusion:**

These data suggest that the chance of a EBLV spill-over from bat to fox is low, but with a greater probability for EBLV-1 than for EBLV-2 and that foxes seem to be able to clear the virus before it reaches the brain and cause a lethal infection.

## Background

Rabies is a viral zoonosis that causes progressive and incurable encephalitis. Rabies infection is caused by neurotropic RNA viruses belonging to the *Rhabdoviridae *family, *Lyssavirus *genus. There are seven classified members of Lyssavirus [1], the classical rabies virus (genotype 1) and the rabies-related viruses (genotypes 2 to 7), with four additional viruses identified more recently in bat species from Europe and Asia: Aravan, Khujand, Irkut and West Caucasian bat viruses [2,3]-these 4 isolates are proposed as new genotypes.

Genotypes (gt) are associated in two immunopathologically and genetically distinct phylogroups [4]. Phylogroup 1 comprises five genotypes: Duvenhage virus (gt 4, Africa), European Bat Lyssaviruses (EBLV-1 (gt 5, Europe), EBLV-2 (gt 6, Europe), Australian Bat Lyssavirus (ABLV) (gt 7, Australia) and the classical rabies virus (gt 1, RABV). Classical rabies viruses circulate in Carnivora world-wide and specifically in the Americas in Chiroptera. Phylogroup 2 includes two African genotypes, Mokola virus (gt 3) and Lagos bat virus (gt 2) isolated from shrews, cats and frugivorous and insectivorous bats. Members of classical rabies virus are found worldwide in Carnivora (both domestic and wild) and in Chiroptera in the Americas), ABLV in frugivorous and insectivorous bats (Australia) while the following genotypes Duvenhage (Africa), EBLV-1 and 2 (Europe) are isolated in insectivorous bats. It has been shown [4], that genotypes of phylogroup 1 are pathogenic for mice when injected by intracranial and intramuscular routes, while Lyssaviruses from phylogroup 2 are less pathogenic by the intramuscular route.

In Europe, bats are important Lyssavirus reservoirs, with more than 800 reported cases since 1954. Out of these 800 European rabid bats, 256 cases have been reported in the Netherlands from 1984 to 2003 [5] and 187 in Germany from 1954 to 2005 [6]. Bats infected with EBLV-1 and EBLV-2 have been reported in several European countries, from Russia to Spain, particularly in coastal regions and more than 95% of the rabid bats are identified as *Eptesicus serotinus*. All infections in *Eptesicus serotinus *are due to the two EBLV-1 subtypes (EBLV-1a and EBLV-1b) [7], while the EBLV-2 subtypes are host-restricted to *Myotis *species. Despite four fatal infections in man (and 3 non-confirmed cases), European Bat Lyssaviruses have rarely been reported to cross the species barrier: dead-end infections have been reported in Denmark in sheep [8,9] and in a stone marten in Germany [10]. Antibodies have also been reported from a cat in Denmark [11], suggesting evidence of infection and more recently a cat from Northwestern France was reported with rabies caused by infection with EBLV-1 [12].

Since the late 1930's, in Europe, red foxes have been the main reservoir and vector of classical rabies. Rabies virus was shown to be highly pathogenic in the fox with an incubation period varying from 11 days to 15 months depending on the dose and on the route of inoculation [13]. Comparative experimental studies on the pathogenicity and on the transmission of rabies have been undertaken using classical rabies virus and EBLVs on various animal models: bats [14-16], mice [17-20], sheep and fox [20-22], and ferrets [23]. The knowledge on the susceptibility of terrestrial, wild and domestic animals to EBLV-1 and EBLV-2 remains limited.

In this study, we have undertaken experimental infections of silver foxes (that belong to *Vulpes vulpes *species, i.e. the same as red fox) by different routes and with different viral doses. The sensitivity of silver foxes to rabies virus isolated from naturally infected foxes has been experimentally shown to be similar for red foxes (M. Artois *et al*., unpublished data).

The presence of the virus in different organs was assessed using the fluorescent antibody test (FAT) and the rabies tissue-culture infection test (RTCIT) [24-26] and reverse transcriptase – polymerase chain reaction (RT-PCR) [27]. The presence of viral RNA on oral swabs was assessed by RT-PCR. Specific neutralizing antibodies in serum samples were measured with classical and modified FAVNt.

## Results

### 1^st ^Clinical diseases in "infected" foxes and results of pathogenicity study

Experimental infection was conducted in several groups of foxes (n = 5) according to virus strains, doses and routes of infection. The outcome of infection for all infected foxes in the five groups is presented in Table 1.

#### EBLV-1 and -2 IC pathogenicity experiment

- For the intracranial experiments using EBLV-1 and EBLV-2, two trials were undertaken. In the first one, two groups of two animals received 4.7 logs MIC LD_50 _of EBLV-1 and 3.2 logs of EBLV-2 respectively (group 2). In the second trial, two animals were infected with 4.4 logs of EBLV-1 and 2.8 logs of EBLV-2 (group 5). (Additional file 1) One negative control was included in that IC experiment.

All foxes in group 2, IC inoculated with either EBLV-1 or EBLV-2, died between 8 and 10 days post-inoculation (p.i.) and between 12 and 22 days p.i. respectively.

The animals showed clinical signs between 2 and 4 days before euthanasia following EBLV-2 inoculation and between 1 and 5 days following EBLV-1 inoculation. The observed clinical signs were the following:

• EBLV-1 (fox 5FD-68AA receiving 4.7 logs MIC LD_50_), dead 8 days p.i., dazed;

• EBLV-1 (fox 5FD-5F3C, 4.7 logs), dead 10 days p.i., loss of appetite, anorexia, hypersalivation;

• EBLV-2 (fox 601-15A3, 3.2 logs), dead 12 days p.i., loss of appetite, anorexia, excitement, crisis;

• EBLV-2 (fox 601-E233, 3.2 logs), dead 22 days p.i., excitement, biting and very aggressive behaviour.

- In group 5, fox 5FE-5AEC inoculated with EBLV-1 (4.4 logs) died 8 days p.i following a phase of paralysis, fox 5FE-58DA inoculated with EBLV-2 (2.8 logs) died 282 days later, showing paralysis and alopecia.

Both antigen and infectious particles were detected by FAT and by RTCIT in brain samples (cortex, hippocampus, cerebellum and medulla oblongata) from all infected animals (5FD-68AA, 5FD-5F3C, 601-15A3 and 601-E233) belonging to the group 2. Viral RNA was detected in the same infected tissues for each animal. Salivary glands were diagnosed rabies positive in foxes 601-E233, 5FE-58DA and 5FD-5F3C inoculated respectively with 3.2 logs and 2.8 logs of EBLV-2 and 4.7 logs of EBLV-1.

IC EBLV-1 and 2 inoculated foxes (5FE-5AEC and 5FE-58DA) belonging to the group 5 were diagnosed positive by FAT (Additional file 1).

#### EBLV-1 and -2 IM pathogenicity experiment

- None of the 5 foxes intramuscularly inoculated with 3.5 logs MIC LD_50 _of EBLV-2 (group 1) died. All foxes remained healthy during the 3-year observation period with no signs indicative of rabies.

Following euthanasia of these five animals, no rabies virus antigen, no infectious particles and no viral RNA were detected in brain or salivary glands samples. None of the five negative controls died during the experiment (except fox numbered 126-DF42 that died of a non-rabid undetermined cause 168 days p.i). All of the controls were lyssavirus negative using referenced diagnosis techniques.

- The doses of EBLV-1 intramuscularly inoculated to the next group of foxes (group 3; n = 6) were distributed as described in Additional file 1.

All six EBLV-1 inoculated foxes, except one (fox 634-8FE1 receiving a viral dose of 3.7 logs MIC LD_50_), survived the challenge till 20 months p.i. and did not develop any clinical signs indicative of rabies or suggestive of encephalitis. The clinical rabies signs observed in fox 634-8FE1 that died 14 days p.i. were: difficulty to swallow, balance problems, dazed appearance. The second fox inoculated with the same dose (fox 633-6AD2) did not exhibit any clinical signs and remained healthy during the 19-month of the observation period. At necropsy of fox 634-8FE1, all specimens of brain (hippocampus, medulla oblongata, thalamus, cortex and cerebellum) were shown to be positive by FAT, RTCIT and RT-PCR. The sub maxillary salivary glands (left and right) were tested negative by FAT. Fox 633-6AD2 was diagnosed negative on brain by FAT, RTCIT and RT-PCR.

- In the third IM experiment (group 4), 3 groups of five animals were infected with serial dilutions of EBLV-1 (4.4, 3.7 and 3 logs MIC LD_50_) as described in Additional file 1. One negative control was included in the assay.

All inoculated foxes, except fox 5FE-45F8 (receiving 3.7 logs of virus), 5FE-60DD (3.0 logs) and 5FD-ACB1 (3.0 logs) survived the challenge and did not develop clinical signs of rabies or signs suggesting encephalitis, during the 13 months of observation. Fox 5FE-45F8 (3.7 logs), had an infected wound on the back, it was euthanized on humane grounds 61 days p.i. It was diagnosed negative by FAT, RTCIT, and RT-PCR.

The clinical signs observed for the two animals 5FE-60DD (3.0 logs) and 5FD-ACB1 (3.0 logs), moribund at 17 and 24 days p.i. were: loss of appetite, anorexia, "strange behaviour evocative of classical rabies" and hypersalivation, prostration, excitement, tendency of biting the cage respectively.

Comparative analysis of FAT and RT-PCR was undertaken on different parts of brain and sub maxillary salivary glands samples collected from foxes 5FE-60DD and 5FD-ACB1 and showed respectively the presence of antigen in medulla oblongata and thalamus, and cortex and thalamus. Viral RNA was shown in the same infected brain tissues and in the following additional tissues: hippocampus, cerebellum and cortex for fox 5FE-60DD and in hippocampus, medulla oblongata, and cerebellum for fox 5FD-ACB1. The sub maxillary salivary glands (left and right) of the two dead animals were rabies negative by FAT and by RT-PCR.

Virus was not isolated from control foxes 126-C920 and 601-F206 that were located in adjacent cages to challenged animals. Following necropsy of these control animals, viral RNA and rabies virus antigen was not detected from brain samples.

### Detection of viral RNA or infectious virus on oral swabs

The confidence of negative PCR results was established by checking for each test the amplification of internal control, 18S rRNA.

All oral swabs taken all over the experiments from foxes inoculated intramuscularly and intracranially with undiluted stock or serially diluted EBLV-1 and EBLV-2 viruses were negative both by RTCIT and by RT-PCR. The fox numbered 5FD68AA, inoculated intracranially with EBLV-1 (EBLV-1 IC group) died 8 days after infection, neither viral RNA nor infectious virus were detected in the oral swab taken one day before the mortality (D7).

Neither viral RNA nor infectious particles were detected on oral swabs taken from "uninoculated" animals during all experiments.

### Rabies virus neutralizing antibodies

The measurement of seroneutralising antibodies has been commonly used for many years to assess the level of protective immunity against rabies in animals. Serological monitoring is used as VNA is the principal modality of protection following rabies infection, which can be correlated with the dose.

In seroneutralisation tests, the dilution of the serum that neutralises 50% of the challenge virus is the efficient dilution 50% (ED_50_). The comparison of this ED_50 _with the one of a reference serum whose titre in IU/ml is established gives the titre of the tested serum. In all the FAVN tests published here, the reference serum used was the OIE international serum of dog origin.

**Figure 1:**
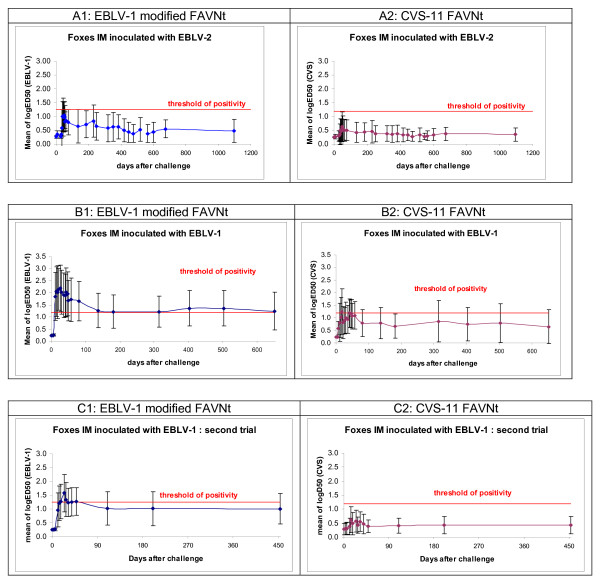
**Kinetics of EBLV-1 and CVS-11 neutralizing activity for foxes intramuscularly infected with EBLV-1 and EBLV-2**. Neutralising activity is expressed by log(ED_50_): dilution giving a 50% neutralization of rabies virus (CVS-11/EBLV-1). Figure 1A: mean of VNA response of 5 foxes intramuscularly inoculated with 10^3.5 ^MIC LD_50 _of EBLV-2 (group 1). The 5 negative controls did not develop any rabies neutralising activity during the entire experiment (log(ED_50_) < 0.24, data not shown). Figure 1B: mean of VNA response obtained in the second trial with intramuscular inoculation of different doses of EBLV-1 (group 3). One fox received 10^4,7 ^MIC LD_50_, one 10^4,4 ^and 2 groups of 2 received respectively 10^3.7 ^and 10^2.7^. One negative control did not develop any rabies neutralising activity all over the experiment (log (ED_50_)< 0.24, data not shown). Figure 1C: VNA response obtained from the fourth IM trial with EBLV-1 (group 4). 3 groups of 5 animals received respectively the final dose of 4.4, 3.7 and 3 logs MIC LD_50_. One negative control fox did not develop rabies neutralising activity all over the entire experiment (log(ED_50_) < 0.24, data not shown). Remark: All neutralising activities are expressed with log(ED_50_) due to the absence of an available specific reference serum of known neutralizing titre against EBLVs.

Figure 1 shows the dynamics of rabies virus neutralizing antibodies of all experimented foxes. Each figure expresses FAVNt results with the neutralising response obtained against either CVS-11 or EBLV-1.

For these serological tests, the log (ED_50_) value of 1.55 corresponds to the specific EBLV-1 titre of 1 UI/ml measured with the OIE dog reference serum that is calibrated against CVS-11. As there are no specific reference serum for EBLVs with a recognized titre (in IU/ml), we have chosen to express the serological results as the log(ED_50_) that corresponds to the neutralization of 50% of the challenge virus.

Several studies on the sensitivity of FAVN test have shown that the threshold of positivity of 0.24 UI/ml could be adopted [28,29]. Based on these previous studies, we assume for this serological study, that animals seroconverted above the value of 1.00 log for ED_50 _in both EBLV-1 mFAVN and CVS-11 FAVNt.

#### EBLV-1 serology

At day 0, all experimented foxes were negative for rabies virus antibody by using both EBLV-1 mFAVNt and CVS-11 FAVNt.

In group 1 (Additional file 1, figure 1A1), all the 5 intramuscularly inoculated animals developed EBLV-2 antibodies. Thirty five days p.i., animal 13C-A15A showed a log(ED_50_) = 1.79. The four remaining animals ranged between 0.24 and 0.36 IU/ml. Four days later (i.e. 39 days p.i.), 4 out of the five inoculated animals showed a rabies EBLV antibody peak (geometric mean = 1.00) with a maximum of 1.67. At the end of the trial (1097 days p.i.) one surviving animal presented a high log(ED_50_) of 1.19 while the 4 others ranged between 0.24 and 0.48.

In group 3 (Additional file 1, figure 1B1), different doses of EBLV-1 were used. Four animals out of the five seroconverted 12 days post infection with log(ED_50_)> 1.00, the mean of log(ED_50_) was 1.85. This value peaked at 2.20 on day 26 p.i. and remained stable up to 81 days p.i. (mean = 1.65). At the end of trial (651 days p.i.), the mean log(ED_50_) was 1.25. Two of the 4 surviving animals demonstrated a log(ED_50_) still positive: 2.15 and 1.91.

In group 4 (Additional file 1, figure 1C1), 14 out of the fifteen animals IM infected with EBLV-1 developed rabies antibody between 11 and 24 days post infection with a mean log(ED_50_) of 0.96 and 1.58 respectively. This value decreased down to 0.44 at the end of trial. One animal (5FD-8E03), receiving 3.7 log of MIC LD_50 _of infectious virus did not develop antibodies after inoculation, all along the experiment, the log(ED_50_) was under 0.24.

Considering that the cut off value for log(ED_50_) is 1.00, 13 animals seroconverted (log(ED_50_) ranging between 1.08 and 2.63) between 11 and 48 days p.i. The 2 foxes that died after inoculation (5FE-60DD and 5FD-ACB1) developed detectable EBLV-1 antibodies before succumbing: log(ED_50_) = 0.96 and 1.91 respectively 14 and 17 days p.i. At the end of the experiment, the mean log(ED_50_) was 1.01 in surviving animals.

Five days after intracranial inoculation with pure EBLV-1 the log(ED_50_) of fox 5FE-5AEC, peaked at 1.08 and then decreased to 0.72 at day 7. The animal was euthanised on day 8 p.i. The other animal intracranially inoculated with EBLV-2 (5FE-58DA) did not develop detectable antibody during the entire experiment, the log(ED_50_) varied between 0.24 and 0.36 between inoculation and 136 days p.i. The animal was euthanased 282 days p.i. with symptoms, without serological analysis of blood samples by FAVNt.

All negative control animals belonging to the five groups remained seronegative with log(ED_50_)<0.24 throughout the experiments.

#### EBLV-2 serology

Foxes inoculated via the IC route did not seroconvert prior to clinical disease during the 22 days of observation (log(ED_50_) = 0.95).

The log(ED_50_) of EBLV-2 specific antibodies of animals inoculated via the IM route generally fluctuated between 1.55 and 2.39, i.e. above the positive threshold (1.43) between days 30 and 330 Of the 5 animals, three had substantially higher antibody levels than the other two.

There were substantial differences between the specific serological responses of the EBLV-2 inoculated animals depending on whether the sera were tested against a homologous genotype (i.e. EBLV-2) or a heterologous one (EBLV-1 or CVS).

#### CVS serology

In group 1 (Additional file 1, figure 1A2), three animals out of the five animals infected with 3.5 log MIC LD_50 _of EBLV-2, seroconverted, log(ED_50_)>1.00 between 28 (13C-A15A: 1.08) and 49 days p.i. (1E2-CB6E: 1.43 and 1E2-D586: 1.67, which is the maximal value observed in this study). The log(ED_50_) of two other animals (13C-F633, 1DF-7D9E) was 0.24 and 0.84. At the end of the trial, all surviving animals (n = 5) presented a log(ED_50_) between 0.24 and 0.48.

Five foxes intramuscularly inoculated with EBLV-1 (group 3, Additional file 1, figure 1B2) seroconverted with log(ED_50_) between 1.08 and 2.03, 12–29 days p.i. The mean value peaked at 18 days p.i. (1.24) and decreased down to 0.65 21 months p.i.

In the final IM EBLV-1 experiment (group 4, Additional file 1, figure 1C2), only 6 animals out of the 15 infected seroconverted above 1.00 between 11 and 39 days post inoculation, with a mean log(ED_50_) varying between 0.38 and 0.47. The mean log(ED_50_) for FAVNt conducted with CVS-11 increased from 0.29 to 0.62 (14-days p.i), then decreased to 0.44 at the end of the trial.

#### Histopathology

**Figure 2:**
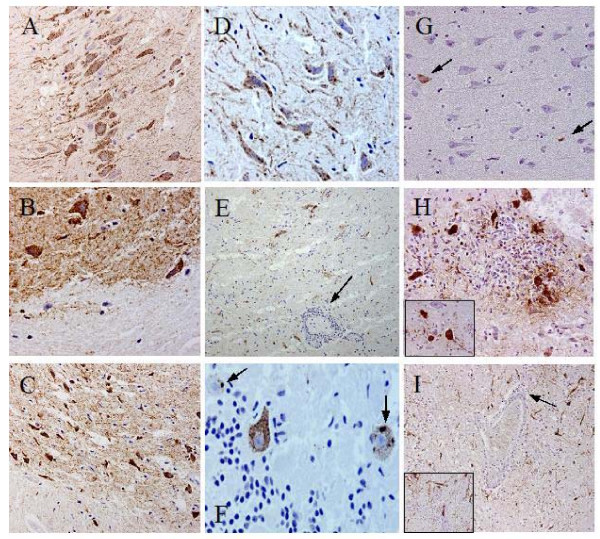
**Immunohistochemistry using the Swiss anti-rabies monoclonal antibody to detect *Lyssavirus *antigen in infected fox brain tissue**. Histopathology legend: Panels A-C* were from an EBLV-1 infected animal inoculated via the IC route. Panels D-F were from an EBLV-2 infected animal inoculated via the IC route. Panels G-I were from an EBLC-1 infected animal inoculated via the IM route. Panels A, D and G were from the hippocampus and panels B, E and H were from the thalmic region. Panel C was from the cortex, panel F from the cerebellum and panel I was from the medulla. Arrows on panels F indicate antigen accumulation reminiscent of Negri bodies, and arrows on panels E and I indicate perivascular, inflammatory infiltrate is also evident in panels E, F, H and I. Arrows on panel G highlight rare positive cells in the hippocampus of an IM inoculated animal. *these sections have a higher 'coffee wash' background as the carcass was frozen prior to post-mortem.

EBLV infected animals were euthanased at a time of advanced disease after which necropsy took place. Both neural and extra-neural tissues were collected for pathology studies. It was observed that brain tissue from IC inoculated foxes demonstrated greater meningoencephalitis or gliosis in the form of generalised and focal cellular infiltrate including peri-vascular cuffing in EBLV-2 infected foxes than EBLV-1 infected animals (Figure 2, panels E, F, H and I) similar to that seen in EBLV-2 infected experimental mice [30]. The distribution and intensity of lyssavirus antigen detection varied between viruses and inoculation route. There was substantially more immunostaining on brain tissue from EBLV-1 infected animals than that from EBLV-2 infected animals following IC inoculation. Regional differences were most prominent in the hippocampus and cerebellum. Comparison of results between IC and IM routes was only possible in the case of EBLV-1 as no animals peripherally inoculated with EBLV-2 succumbed to disease. In the case of EBLV-1 both antigen detection and inflammatory infiltrate varied in regions of the brain. The most obvious differences included the rarity of infected neurons in the hippocampus in IM inoculated animals and enhanced inflammatory infiltrate in the thalamus and medulla.

Cervical spinal cord sections were positive in both IC inoculated EBLV-1 and EBLV-2 infected animals with distinct signal in many (40–50%) motor neuron cell bodies. Thoracic and cervical spinal sections were negative as confirmed by PCR and virus re-isolation (RTCIT). Isolated neurons in the brachial plexi were also positive but this could not be confirmed by other methods. Other extra-neural tissues contained a small number of non-specifically stained cells.

## Discussion

Red foxes are the principal reservoir and vector of classical rabies virus (genotype 1) in Western Europe [31]. Thanks to the campaigns of oral vaccination of foxes, France and other Western European countries have been rabies free for many years [32]. This study was undertaken to assess the risk of spillover of a bat lyssavirus strain from a bat to a fox and causing rabies infection with EBLV-1 and EBLV-2, and to investigate whether or not foxes can transmit EBLVs. These experiments were conducted with high doses of virus using two different routes of infection: intramuscular and intracranial.

In our study, we have shown that all foxes infected experimentally intracranially with undiluted stock or diluted batches of EBLV-1 and 2 exhibited clinical signs of rabies and succumbed between 8 and 282 days post infection. All clinically ill foxes developed neutralising antibodies and contained viral antigen, infectious particles and viral RNA in the brain. Salivary glands were positive in three out of six animals, but none of the oral swabs collected from these animals were positive for viral RNA or infectious particles.

The two successive intramuscular trials undertaken with EBLV-1 (groups 3 and 4) did not give reproducible results, and surprisingly, some foxes challenged with the minimal doses of EBLV-1 succumbed and presented neurological clinical signs indicative of rabies. Surprisingly, in both trials, none of the foxes infected with undiluted stock succumbed, and all but two, developed a high neutralising antibody response suggesting an abortive peripheral infection. Similarly to intracranial experiments, neither viral RNA nor infectious particles were detected in any of the saliva obtained with oral swabs collected regularly from all infected animals and kept dry on ice until analysis.

In our study, we have shown the influence of the strain of challenge virus used in FAVN assays (homologous vs. heterologous). Similar observations have been previously reported by Moore et al [33]. FAVNt assays using homologous virus (EBLV-1) reported higher neutralising titres for all foxes infected intramuscularly with EBLV-1 than when the assay was conducted against CVS-11. This differential neutralising response has also been observed from a qualitative point of view. With the EBLV-1 modified FAVNt, 17 of the 21 experimented animals seroconverted while only 6 seroconverted with FAVNt conducted against CVS-11.

Rabies pathogenesis has been assessed by several groups by using different variants of Lyssavirus including the classical genotype 1 isolated from infected American bats, dogs or foxes [34-36], and EBLV-1 and EBLV-2 on ferrets, sheep, foxes, cats and mice [17,20-23]. More recently, experimental studies in bats using EBLV-1 and EBLV-2 have shown that bats are likely to transmit the virus following a shallow bite to peripheral tissue [15,16].

Infection on mice with EBLV-1 in our experiments involved agitation, hyperactivity and aggressiveness before death (data not shown). Clinical signs observed in mice after infection with EBLV-2 were different from the ones following EBLV-1 infection: prostration and paralysis before death are frequent (VLA, unpublished data). The same variable clinical signs have also been observed in foxes after infection with rabies viruses: experimentally infected foxes first exhibit loss of appetite, hyperactivity, tremor, hypersensitivity, and sudden aggressiveness, convulsions and paralysis are common late signs [37,38]. In our study, we observed that the clinical phase is short and varies between 2 to 6 days. Similar results were shown by some rabies pathologists and reviewed by Wandeler in 2004 [38], the clinical phase lasted one to seventeen days [38,39].

In this study, foxes have been infected intracranially (i.e., non classical route different from previous trial with fox and classical virus strains) and only six animals have been inoculated. The two facts make the comparison of observed clinical signs between EBLVs and genotype 1 virus inconclusive. The only reliable observations undertaken on these foxes are the following: death occurs within one week post inoculation and usual clinical signs of rabies have been observed.

Red foxes have been shown to be highly susceptible to classical rabies by the intramuscular route, for example, the IM LD_50 _of a street rabies virus isolated in North America intramuscularly was less than 5 MIC LD_50 _[40]. In the same way, Blancou et al. [39] showed that the intramuscular route is 12.6 × 10^5 ^to 15.85 × 10^5 ^times more efficient to infect red foxes than the oral route. The dose of virus, the route of inoculation and entry into the central nervous system are of the utmost importance. It has been suggested that both virus and host factors including strain, route of inoculation, distance of inoculation site from the brain and the immunologic status of the host, influence the incubation periods, and whether the resulting infection is abortive or fatal [41].

In our study, we have shown that fatal clinical diseases can be induced by intracranial route, while the intramuscular route and a high dose of EBLV result in a non-fatal disease, accompanied by a serological neutralising response. Similar to our study, it has been shown by Blancou et al. [42] that foxes were susceptible to low doses of canine rabies virus from North Africa, and resisted the inoculation of a high dose and became immune, reflecting a possible abortive peripheral infection or a failure in virus replication.

Spill over of rabies virus (genotype 1) has been reported to occur from bats to red foxes in Newfoundland, Canada [43] and from bats to skunks in Arizona, USA [44]. These observations suggest that North American bat strains of rabies virus more readily spill over and adapt to a new host when compared with EBLVs. We speculate that these observations suggest that 'Old World' bat strains of rabies virus have evolved over thousands of years and are fully-adapted to their chiropteran host. In contrast, we hypothesise that 'New World' bat strains of rabies virus are in evolutionary terms 'younger' viruses, are less adapted to their chiropteran host and are therefore more prone to spill over in search of a new mammalian host.

Because of the narrow co-adaptation between a rabies strain and its usual normal host, spill overs into other species have a very low probability in relation to establishing a new rabies cycle. It means that specific virus variants within a single genotype (fox – dog variants in genotype 1) tend to be maintained in their specific hosts without mixing. As for Carnivora, the compartmentalisation of bat rabies seems to be the epidemiological rule, although spill-over infection to other mammals has been documented [45].

Natural transmission of EBLV-1 to terrestrial animals has been previously reported in Danish sheep [8,9] and in a stone marten in Germany [10], the first case of transmission in terrestrial wildlife. Ferrets have been reported by Vos et al. [23] as being susceptible to infection with a high dose of EBLV-1 (between 10^4 ^to 10^6 ^FFU), resulting in an incubation period of between 7 and 15 days. These animals are dead-end hosts and are unable to transmit the infectious virus to a new host. Similarly to our results, all ferrets that became clinically ill were positive by FAT in brain, all oral swabs were negative and viral RNA could only be detected in salivary glands of infected animals [23].

It has rarely been reported that infection of mammals with EBLV has resulted in fatal disease [22]. Soria Balthazar et al. [46] infected sheep with an EBLV-1 virus isolated from a rabid bat (*Eptesicus serotinus*) in Denmark in 1985 (originally identified as "Duvenhage like" virus), with doses ranging between 10^5.6 ^and 10^4.6 ^MIC LD_50_. Only one animal infected with the highest dose succumbed to disease 30 days post inoculation, the incubation period lasted 8 days. No antigen was detected in the brain while infectious particles were detected by mouse inoculation test. Additionally, it has been shown in the same study, that 3 out of 5 foxes intramuscularly inoculated into the masseter with different doses of "Duvenhage like" virus, died between 11 and 20 days post infection [46]. Rabies diagnosis (FAT, Negri bodies and mouse inoculation test) undertaken on the brains of these 3 animals showed the presence of rabies antigen in two and infectious particles have been detected in one.

All of these experiments, including our results, strongly suggest that the outcome of disease after exposure to EBLVs may be dependent on the host species, the inoculation site and the type of infecting virus.

Most natural rabies cases result from the bite of a rabid animal [46], although infection through aerosols under experimental conditions in mice has been reported [19]. The other routes of infection are rare. Few studies have been undertaken to monitor the viral shedding of naturally or experimentally infected animals. Bats naturally or experimentally infected with genotype 1 Lyssavirus have been shown to harbour virus in the nasal mucosa [47] and in the saliva (*T. brasiliensis*, [48]) respectively, leading to the supposition that a transmission between bats living in the same colony is possible. Recently Hughes et al. [49] reported the presence of viral RNA in oral swabs of experimented bats infected with Khujand and Irkut new Lyssaviruses, between 0 and 2 days before the onset of illness. In 2006, the presence of viral RNA has been detected by molecular tools on an oral swab collected from a common serotine naturally infected with EBLV-1 three days before death; no infectious virus was detected. Our experiments have not detected viral RNA or virus infectious particles in any of the oral swabs collected throughout the experiments. As observed in other infection models, shedding of virus may be intermittent and can explain the absence of detection of viral RNA in oral swabs. Comparative studies should be undertaken in order to further ameliorate our understanding of rabies virus excretion in saliva and salivary gland infection.

The diseased brain tissue was characterised by diffuse meningioencephalitis with gliosis and perivascular cuffing as observed in EBLV-1 infected cats and dogs [17] and viral antigen is seen in neurons throughout the various regions of the brain. There was a suggestion of antigen distribution differences in the cerebellum and hippocampus; the signal appeared less intense and more focal in EBLV-2 compared to that in EBLV-1 infected tissue. In general the number of infected cells and intensity of staining was greater for EBLV-1 than EBLV-2, inclusion bodies similar but less distinct than RABV Negri bodies were observed in EBLV-2 infected cells but rarely in EBLV-1 infected tissue, and inflammation was more prevalent in EBLV-2 than EBLV-1. Inflammation of the brain in the form of gliosis and perivascular cuffing were also observed in those foxes that succumb to clinical disease; EBLV-1 IC and IM and EBLV-2 IC inoculations.

Similar observations recently conducted by Brookes et al. [22] on IC and IM sheep experiments with EBLV-1 and-2, showed that as the previous study conducted on ferrets [23], EBLVs show a limited capacity to induce lethal disease when inoculated at a peripheral site (IM route).

The pathogenicity of EBLV-1 for foxes appears to be low using the intramuscular route, which closely mimics the classical natural transmission route of rabies virus to new hosts. Furthermore, the absence of detection of infectious particles or of viral RNA in saliva throughout the intracranial and intramuscular experiments undertaken in our study suggests that foxes may not be able to transmit EBLV-1 as virus was not detected in the saliva of clinically infected animals. The chance of EBLV spill over from bat to fox is low, there is a minimal chance of contact between insectivorous bats and foxes which seem to be dead-end hosts for EBLVs viruses.

## Conclusion

The susceptibility of foxes to experimental infection with EBLVs appears to be very low and thus the virus represents a low risk to red foxes in the population. This does not, however, negate the fact that red foxes are the main host and vector of classical rabies in Europe. Further studies, including the use of different EBLV isolates and doses, different routes and sites of infection and the use of other potential host species as experimental models are required in order to ameliorate our understanding of pathogen adaptation and of the host response, which can result in an abortive infection, survival with clinical sequelae or lethal infection. Further *in vitro *studies on the EBLV-1 neurotropism and similar fox experiments with the subcutaneous route to mimic superficial exposure should be undertaken to assess the risk of EBLV spillover.

## Methods

### Animals

The captive bred silver foxes used in the study were purchased from the breeding centre of the Norvegian Fur Breeder's Association (Oekern, 0509, Oslo, Norway). These animals belong to the same zoological species as the red fox, *Vulpes vulpes*. All animals were individually identified with microchips. They were kept for observation in individual cages. They were fed daily with commercial food for dogs (PR27 Royal Canin, France) and watered *ad libitum*. Animals were clinically observed once or more daily and the general behaviour of each animal was recorded by two trained personnel.

### Viruses

EBLV-1b and EBLV-2b strains were passaged 3 times intracranially in mice from the original bat brain. A 10% brain suspension was prepared in DMEM and clarified by centrifugation at 1500 g at 4°C for 30 minutes. The viral stocks were stored in liquid nitrogen in 0.75 ml aliquots having a viral titre of 5.4 log MIC LD_50_/ml for EBLV-1 and 4.2 log MIC LD_50_/ml for EBLV-2 stock. These stocks constituted the challenge batch used for all experimental inoculations. Back titration in mice was undertaken after each experiment to confirm the inoculated dose.

EBLV-1 virus was isolated from the brain of a naturally rabid serotine bat (*Eptesicus serotinus*) found in Lurcy Levis in the Centre of France in 2002 (animal registered under no:123008 in AFSSA Nancy, France). The challenge strain of EBLV-2 was prepared from an infected *Myotis daubentonii *from Lancashire in United Kingdom [50] (animal registered under no:RV1332, VLA, United Kingdom).

### Experimental infections

Three studies were conducted according to instructions related to animal experimentation provided by the French Ethical Committee using EBLV-1 and EBLV-2 viruses in 40 animals. Both intramuscular and the intracranial routes were used. Additional file 1 summarises the experimental details (identification of foxes, route of inoculation, volume injected, dose and type of virus inoculated, viral dose expressed in LD_50_/mouse).

The intramuscular injection was administered deep in the left temporal muscle with a volume of 1 ml of viral suspension on vigil animals. The intracranial injection was undertaken with a volume of 0.5 ml on anaesthetized foxes (0.85 mg/kg medetomidine plus 100 mg/kg ketamine IM). The frontal bone was then trephined 1 cm behind the insertion of temporalis muscle and 2 cm left of the sagital plan. The injection was given slowly with a 0.6 mm in diameter needle introduced 20 mm in the hole. After the skin suture, the recovery product (atipamezole 4.28 mg/kg, IM) was injected and a preventive antibiotic treatment was conducted (amoxicillin, 15 mg/kg SC) for 5 days. Using this procedure, the injection site was supposed to be roughly identical to the one used in mice, i.e. in the first third of the cortex.

### Clinical observations and sampling

Before the inoculation of foxes, serum and saliva samples were collected from all 40 animals and stored at 4°C until examined for neutralizing antibodies and rabies virus using the cell culture test and by PCR as described below.

The first group of animals included 5 EBLV-2 inoculated (IM) animals and 5 negative controls, serum and saliva samples were regularly collected once a week during 2 months, then on a monthly base over 2 years, i.e. on days 0, 7, 28, 32, 35, 38, 42, 46, 49, 53, 56, 63, 77, 137, 189, 230, 250, 320, 351, 383, 418, 446, 474, 518, 546, 565, 600, 676 & 1097. The second group included 4 animals inoculated intracranially with EBLV-2 and one control, samples of serum and saliva were collected 7 days post-infection. All animals in group 3 (6 EBLV-1 inoculated intramuscularly, 1 negative control) and group 5 (1 EBLV-1 intracranially inoculated and 1 EBLV-2 intracranially inoculated), had both serum and saliva samples collected on days 0, 5, 7, 12, 14, 18, 22, 26, 29, 36, 41, 43, 47, 50, 57, 81, 136, 181, 314, 404, 503 and 651. The last group of animals (15 EBLV-1 foxes intramuscularly inoculated with varying inoculation doses and 5 uninoculated controls) samples were collected on the following days: 0, 3, 6, 11, 14, 18, 24, 28, 32, 39, 48, 110, 200 & 454.

### Rabies diagnosis, Molecular biology applied to rabies virus, serology and related procedures

After humane euthanasia of animals (either those having demonstrated clinical signs, humane end-points or at the end of the experiment) with intravenous T61 (Intervet, France), samples were collected from the brain (left and right areas of the hippocampus, portions of medulla oblongata, portions of thalamus, portions of cortex and cerebellum) and from sub-maxillary salivary glands. These samples were then analysed to detect for the presence of antigen (FAT), infectious virus (RTCIT) and viral RNA (VPCR), as described below.

### Fluorescent antibody test: FAT

The FAT was undertaken as previously described [24]. Briefly, impression smears from the eight different areas of the brain and salivary glands were undertaken, air-dried, fixed in cold acetone at -20°C for 30 minutes and stained with a polyclonal fluorescein isothiocyanate labelled rabbit anti-rabies virus nucleocapsid immunoglobulin (BIORAD, France). The slides were then read under a fluorescent microscope by two trained laboratory personnel.

### Histopathology

Tissue samples from all animals were fixed in 4% phosphate-buffered formaldehyde and processed for paraffin-embedding. Paraffin-wax sections (3 μm) were dewaxed and stained with haematoxylin and eosin. To analyze distribution of EBLV antigens within the brain, sections were mounted on SuperFrost^® ^plus microscope slides, dewaxed and rehydrated as described by Brookes et al. [22]. The sections were incubated with a mouse monoclonal antibody against the lyssaviral nucleoprotein antigen (Swiss MAB-HAM, kindly provided by the Swiss Rabies Centre) at a dilution of 1:800 in Tris-buffered-saline (TBS, 0.1 M Tris-base, 0.9% NaCl, pH 7.6). A biotinylated goat anti-mouse IgG1 (Vector, Burlingame, CA) was used as linker-antibody for the avidin-biotin-complex (ABC-) method. A nonspecific primary antibody (source of isotype control) and mock infected control mouse tissue acted as negative controls and were analysed in parallel. By means of the ABC-method and an immunoperoxidase kit (Vectastain Elite ABC Kit, Vector), a brown-red signal was obtained from the substrate 3-amino-9-ethylcarbazole (DAKO AEC substrate-chromogen system; Dako, Carpinteria, CA, USA). The sections were counterstained with Mayer's haematoxylin, and sealed with aqueous medium (Aquatex; Merck, Darmstadt, Germany).

### Cell culture inoculation test: RTCIT

The test was undertaken using a modified procedure as described by Barrat et al. [26] in eight chamber labtek slides. Ten percent (w/v) tissue suspensions were prepared using DMEM containing antibiotics and 50% NBCS and centrifuged at 1500 g for 10 minutes. A volume of 50 μL of supernatant from each sample (brain, salivary glands) was then added to individual wells containing 0.4 × 10^5 ^neuroblastoma cells in 400 μl. Following incubation at 35°C (with 5% CO_2_) for 48 hours, the medium was changed 1 day later, on day 2 cell culture medium was discarded and the slides were fixed in cold acetone, stained and examined as described for the FAT.

In the same manner, the cell culture test was undertaken on the pre-weighted oral swabs which had been kept dry on ice till the end of sampling. The swabs were 1:10 (m:v) diluted with medium culture supplemented with antibiotics, centrifuged at 4000 g for 30 minutes and the swabs were discarded. The eluate was stored at +4°C after centrifugation then tested by cell culture test and by RT-PCR before freezing at -80°C. Positive (CVS-27 and EBLV-1) and negative (cell culture medium) controls were incorporated in each test.

### RNA extraction and RT-PCR

Viral RNA was extracted from 150 μL of either 10% tissue suspensions supernatant (different areas of brain and salivary glands) or from swab medium using Qiagen Viral RNA mini kit according to the manufacturer's instructions. First and second round polymerase chain reactions were carried out as described by Heaton et al. [51] Host RNA control (18S rRNA) was amplified for each sample with RT-PCR according to the technique previously described by Smith et al. [52].

### Controls

To avoid false-positive PCR results, the usual precautions for PCR were strictly followed in the laboratory [53]. Amplification of 18S rRNA was undertaken for each sample to control the integrity of RNA and validate each negative result of RT-PCR. All samples were then analysed twice: once for host rRNA (18S rRNA,) and the second with lyssavirus universal primers [51].

### Serology

The detection of EBLV-1, EBLV-2 and CVS-11 specific neutralising antibodies in the serum samples collected from foxes was carried out by using the modified and standard fluorescent antibody virus neutralization test (mFAVN, FAVNt) [54,55]. EBLV-2 mFAVN was undertaken at the VLA laboratory, FAVNt with CVS-11 and EBLV-1 mFAVN were undertaken at the AFSSA laboratory.

Briefly, duplicate or quadruplicate serum samples, with a volume of 50 μl, were diluted, added to each well, incubated at 37°C for 1 hour with around 100 TCID_50 _of specific virus: CVS-11, acting as positive control and EBLV-1 or EBLV-2. A volume of 50 μl of BHK-21 cells suspension was added in each well and left for two days at 37°C, before staining with fluorescein-isothiocyanate anti-rabies monoclonal antibody (Centocor INC, Malvern, Pennsylvania, USA). Controls included uninfected BHK-21 cells, OIE positive serum, negative serum from dog and titration of specific virus (CVS-11, EBLV-1). And rabbit anti-EBLV-1 and EBLV-2 for performing EBLV-2 modified FAVNt.

An additional positive control for FAVNt was carried out by using CVS-11. VNAs were expressed in log ED_50 _giving a 50% neutralization of rabies virus (around 100 TCID_50 _of CVS-11, EBLV-1 and EBLV-2) the reference material for EBLV-1 neutralizing antibodies detection being not available for the experiments.

The threshold of positivity was established to 1.19 log ED_50 _for CVS-11, 1.25 log ED_50 _for EBLV-1 and 1.43 for EBLV-2.

## Authors' contributions

DH, EL, MB and LJ participated in sample harvest and carried out the laboratory work. SB and JB participated in discussion of the project, technical support and contributed to the manuscript preparation. MW supervised serological activities, analysis of serological data and participated in the manuscript preparation. EP participated in discussion of the project, technical support, performance of assay, analysis of data and manuscript preparation. FC and TF designed, coordinated and supervised all the study, analysis of data and manuscript preparation. All authors read and approved the final manuscript.

## Supplementary Material

Additional File 1 **Table 1 a and b**. Details and outcome of the experimental infections (FAT, RTCIT and RT-PCR results on brain tissues collected from cadavers after sacrifice) undertaken on foxes with EBLV-1 and EBLV-2 viruses. Click here for file
