# Supplementary material for: Experimental infection of Foxes with European bat Lyssaviruses type-1 and 2
Source: BMC Vet Res. 2009 May 19;5:19. doi: 10.1186/1746-6148-5-19 (PMC2694770; doi:10.1186/1746-6148-5-19)
Supplement: Additional File 1 — Table 1 a and b. Details and outcome of the experimental infections (FAT, RTCIT and RT-PCR results on brain tissues collected from cadavers after sacrifice) undertaken on foxes with EBLV-1 and EBLV-2 viruses. [file 1746-6148-5-19-S1.doc]

| **Inoculated Virus** | **Fox microchip** | **Sex** | **Age of animal when inoculated**  **(year)** | **Status** | **Route of inoculation** | **Group** | **Log (inoculated dose) MIC LD_50_** | **Outcome of infection** | **Conditions of sacrifice** | **Day of mortality** | **Clinical phase**  **(days)** |
| --- | --- | --- | --- | --- | --- | --- | --- | --- | --- | --- | --- |
|  |  |  |  |  |  |  |  |  |  |  |  |
| EBLV-2  IC group | 5FF-EC0E | F | 1 | NC | / | 2 | / | E* | Sacrificed “healthy” | D134 | - |
|  | 601-E233 /  5FE-4400 | F | 1 | I | IC | 2 | 3.2 | E | Sacrificed with symptoms | D22 | 5 |
|  | 601-15A3 | F | 1 | I | IC | 2 | 3.2 | E | Sacrificed with symptoms | D12 | 3 |
| EBLV-1  IC group | 5FD-68AA | F | 1 | I | IC | 2 | 4.7 | E | Sacrificed with symptoms | D8 | 2 |
|  | 5FD-5F3C | F | 1 | I | IC | 2 | 4.7 | E | Sacrificed with symptoms | D10 | 6 |
| EBLV-1 IC fox | 5FE-5AEC | F | 3 | I | IC | 5 | 4.4 | E | Sacrificed with symptoms | D10 | 2 |
| EBLV-2 IC fox | 5FE-58DA | F | 3 | I | IC | 5 | 2.8 | E | Sacrificed with symptoms | D282 | No observation |
| EBLV-2  IM group | 1BF-05C3 | F | 3 | NC | / | 1 | / | S |  |  |  |
|  | 5FD-9E54 | M | 3 | NC | / | 1 | / | S |  |  |  |
|  | 126-DF42 | M | 3 | NC | / | 1 | / | E* | Sacrificed “healthy” | D168 | - |
|  | 1DA-73C3 | M | 6 | NC | / | 1 | / | S |  |  |  |
|  | 1DF-815D | M | 3 | NC | / | 1 | / | S |  |  |  |
|  | 1E2-D586 | F | 6 | I | IM | 1 | 3.5 | S |  |  |  |
|  | 1E2-CB6E / 1E2-D504 | F | 5 | I | IM | 1 | 3.5 | S |  |  |  |
|  | 13CA15A | F | 6 | I | IM | 1 | 3.5 | S |  |  |  |
|  | 1DF-7D9E | F | 3 | I | IM | 1 | 3.5 | S |  |  |  |
|  | 13CF633 | F | 6 | I | IM | 1 | 3.5 | S |  |  |  |
| First  EBLV-1  IM group | 601-F206 | M | 1 | NC | / | 3 | / | S |  |  |  |
|  | 634-95B9 | F | 1 | I | IM | 3 | 2.7 | S |  |  |  |
|  | 646-77E4 | M | 1 | I | IM | 3 | 2.7 | S |  |  |  |
|  | 634-8FE1 | F | 1 | I | IM | 3 | 3.7 | E | Sacrificed with symptoms | D14 | 2 |
|  | 633-6AD2 | M | 1 | I | IM | 3 | 3.7 | S |  |  |  |
|  | 634-A142 | M | 1 | I | IM | 3 | 4.7 | S |  |  |  |
|  | 646-18FF | M | 1 | I | IM | 3 | 4.4 | S |  |  |  |
| Second  EBLV-1  IM group | 126-C920 | F | 5 | NC | / | 4 | / | S |  |  |  |
|  | 5FD-64C6 | M | 3 | I | IM | 4 | 4.4 | S |  |  |  |
|  | 601-E9B5 | F | 3 | I | IM | 4 | 4.4 | S |  |  |  |
|  | 125-804D | F | 4 | I | IM | 4 | 4.4 | S |  |  |  |
|  | 5FF-F020 | F | 3 | I | IM | 4 | 4.4 | S |  |  |  |
|  | 5FE-518C | F | 3 | I | IM | 4 | 4.4 | S |  |  |  |
|  | 5FD-8E03 | F | 3 | I | IM | 4 | 3.7 | S |  |  |  |
|  | 5FE-45F8 | F | 3 | I | IM | 4 | 3.7 | E * | Sacrificed „healthy“ | D61 | - |
|  | 5FC-A399 | M | 3 | I | IM | 4 | 3.7 | S |  |  |  |
|  | 5FE-3F34 | F | 3 | I | IM | 4 | 3.7 | S |  |  |  |
|  | 5FE-57AB | F | 3 | I | IM | 4 | 3.7 | S |  |  |  |
|  | 601-CE5E / 600-C38A | F | 3 | I | IM | 4 | 3 | S |  |  |  |
|  | 5FE-60DD | M | 3 | I | IM | 4 | 3 | E | Sacrificed with symptoms | D17 | 4 |
|  | 5FD-ACB1 | F | 3 | I | IM | 4 | 3 | E | Sacrificed with symptoms | D24 | 5 |
|  | 5FF-A896 | M | 3 | I | IM | 4 | 3 | S |  |  |  |
|  | 601-DBF4 | F | 3 | I | IM | 4 | 3 | S |  |  |  |

|  |  |  | **Presence in brain of** | | | **Presence in salivary glands of** | | | **Presence in oral swabs of** | |
| --- | --- | --- | --- | --- | --- | --- | --- | --- | --- | --- |
| **Inoculated Virus** | **Fox microchip** | **Group** | **Antigen** | **Infectious Particles** | **Viral**  **RNA** | **Antigen** | **Infectious Particles** | **Viral**  **RNA** | **Infectious Particles** | **Viral**  **RNA** |
| EBLV-2  IC group | 5FF-EC0E | 2 | neg | neg | neg |  |  |  | neg | neg |
|  | 601-E233 /  5FE-4400 | 2 | **pos** | **pos** | **pos** | **pos** | **neg** | **pos** | neg | neg |
|  | 601-15A3 | 2 | **pos** | **pos** | **pos** | neg | neg | neg | neg | neg |
| EBLV-1  IC group | 5FD-68AA | 2 | **pos** | **pos** | **pos** | neg | Neg | neg | neg | neg |
|  | 5FD-5F3C | 2 | **pos** | **pos** | **pos** | **pos** | **pos** | **pos** | neg | neg |
| EBLV-1 IC fox | 5FE-5AEC | 5 | **pos** | NP | NP | neg | neg | ND | neg | neg |
| EBLV-2 IC fox | 5FE-58DA | 5 | **pos** | **pos** | **pos** | **pos** | **pos** | ND | neg | neg |
| EBLV-2  IM group | 1BF-05C3 | 1 | neg | neg | neg |  |  |  | neg | neg |
|  | 5FD-9E54 | 1 | neg | neg | neg |  |  |  | neg | neg |
|  | 126-DF42 | 1 | neg | neg | neg |  |  |  | neg | neg |
|  | 1DA-73C3 | 1 | neg | neg | neg |  |  |  | neg | neg |
|  | 1DF-815D | 1 | neg | neg | neg |  |  |  | neg | neg |
|  | 1E2-D586 | 1 | neg | neg | neg |  |  |  | neg | neg |
|  | 1E2-CB6E / 1E2-D504 | 1 | neg | neg | neg |  |  |  | neg | neg |
|  | 13CA15A | 1 | neg | neg | neg |  |  |  | neg | neg |
|  | 1DF-7D9E | 1 | neg | neg | neg |  |  |  | neg | neg |
|  | 13CF633 | 1 | neg | neg | neg |  |  |  | neg | neg |
| First  EBLV-1  IM group | 601-F206 | 3 | neg | neg | neg |  |  |  | neg | neg |
|  | 634-95B9 | 3 | neg | neg | neg |  |  |  | neg | neg |
|  | 646-77E4 | 3 | neg | neg | neg |  |  |  | neg | neg |
|  | 634-8FE1 | 3 | **pos** | **pos** | **pos** | neg | NP | NP | neg | neg |
|  | 633-6AD2 | 3 | neg | neg | neg |  |  |  | neg | neg |
|  | 634-A142 | 3 | neg | neg | neg |  |  |  | neg | neg |
|  | 646-18FF | 3 | neg | neg | neg |  |  |  | neg | neg |
| Second  EBLV-1  IM group | 126-C920 | 4 | neg | neg | neg |  |  |  | neg | neg |
|  | 5FD-64C6 | 4 | neg | neg | neg |  |  |  | neg | neg |
|  | 601-E9B5 | 4 | neg | neg | neg |  |  |  | neg | neg |
|  | 125-804D | 4 | neg | neg | neg |  |  |  | neg | neg |
|  | 5FF-F020 | 4 | neg | neg | neg |  |  |  | neg | neg |
|  | 5FE-518C | 4 | neg | neg | neg |  |  |  | neg | neg |
|  | 5FD-8E03 | 4 | neg | neg | neg |  |  |  | neg | neg |
|  | 5FE-45F8 | 4 | neg | neg | neg |  |  |  | neg | neg |
|  | 5FC-A399 | 4 | neg | neg | neg |  |  |  | neg | neg |
|  | 5FE-3F34 | 4 | neg | neg | neg |  |  |  | neg | neg |
|  | 5FE-57AB | 4 | neg | neg | neg |  |  |  | neg | neg |
|  | 601-CE5E / 600-C38A | 4 | neg | neg | neg |  |  |  | neg | neg |
|  | 5FE-60DD | 4 | **pos** | **pos** | **pos** | neg | neg | neg | neg | neg |
|  | 5FD-ACB1 | 4 | **pos** | **pos** | **pos** | neg | neg | neg | neg | neg |
|  | 5FF-A896 | 4 | neg | neg | neg |  |  |  | neg | neg |
|  | 601-DBF4 | 4 | neg | neg | neg |  |  |  | neg | neg |
|  |  |  |  |  |  |  |  |  |  |  |

Abbreviations:

I: inoculated animal, NC: negative control, IC, intracranial infection; IM, intramuscular infection; M: male; F: female; S: survival; E: euthanasia of rabid animals; NP: not performed.

E* : After death, the detection of rabies virus was undertaken on brain by rabies diagnosis and molecular tools. No infectious rabies virus, antigen or viral RNA was detected in these tested tissue. Titre of inoculated dose is expressed in log (MIC LD_50_).

Foxes either survived (S) until the end of the trial or were euthanised (E) on the day Di (in brackets), with or without rabies clinical signs, as indicated above.

Surviving foxes were euthanized between day 1097 and 1113 day p.i. for the EBLV-2 IM inoculated group, on day 651 p.i. for the first EBLV-1 IM inoculated group and between day 447 and day 454 p.i. for the second EBLV-1 IM inoculated group.
